# Distinct Roles of the Phosphatidate Phosphatases Lipin 1 and 2 during Adipogenesis and Lipid Droplet Biogenesis in 3T3-L1 Cells*

**DOI:** 10.1074/jbc.M113.488445

**Published:** 2013-10-16

**Authors:** Hiroshi Sembongi, Merce Miranda, Gil-Soo Han, Stylianos Fakas, Neil Grimsey, Joan Vendrell, George M. Carman, Symeon Siniossoglou

**Affiliations:** From the ‡ Cambridge Institute for Medical Research, University of Cambridge, CB2 0XY Cambridge, United Kingdom,; the § Centro de Investigación Biomédica en Red de Diabetes y Enfermedades Metabólicas Asociadas, Institut d'Investigació Pere Virgili, Universitat Rovira i Virgili, Hospital Universitari Joan XXIII, Tarragona, Spain, and; the ¶ Department of Food Science, Rutgers Center for Lipid Research, and New Jersey Institute for Food, Nutrition and Health, Rutgers University, New Brunswick, New Jersey 08901

**Keywords:** Adipocyte, Lipids, Mouse, Phosphatase, Phosphatidate, Triacylglycerol, Lipin

## Abstract

Lipins are evolutionarily conserved Mg^2+^-dependent phosphatidate phosphatase (PAP) enzymes with essential roles in lipid biosynthesis. Mammals express three paralogues: lipins 1, 2, and 3. Loss of lipin 1 in mice inhibits adipogenesis at an early stage of differentiation and results in a lipodystrophic phenotype. The role of lipins at later stages of adipogenesis, when cells initiate the formation of lipid droplets, is less well characterized. We found that depletion of lipin 1, after the initiation of differentiation in 3T3-L1 cells but before the loading of lipid droplets with triacylglycerol, results in a reciprocal increase of lipin 2, but not lipin 3. We generated 3T3-L1 cells where total lipin protein and PAP activity levels are down-regulated by the combined depletion of lipins 1 and 2 at day 4 of differentiation. These cells still accumulated triacylglycerol but displayed a striking fragmentation of lipid droplets without significantly affecting their total volume per cell. This was due to the lack of the PAP activity of lipin 1 in adipocytes after day 4 of differentiation, whereas depletion of lipin 2 led to an increase of lipid droplet volume per cell. We propose that in addition to their roles during early adipogenesis, lipins also have a role in lipid droplet biogenesis.

## Teaser

**Background:** Lipins are phosphatidate phosphatases that generate diacylglycerol for lipid synthesis.

**Results:** Lipin 1 or lipin 2 depletion has distinct effects on differentiating adipocytes. Cells depleted of both lipins after initiation of adipogenesis accumulate triacylglycerol but display lipid droplet fragmentation.

**Conclusion:** Lipins have a role in lipid droplet biogenesis after initiation of adipogenesis.

**Significance:** Lipins play multiple roles during adipocyte differentiation.

## Introduction

Lipins define a family of Mg^2+^-dependent PA^3^ phosphatase enzymes with key roles in lipid metabolism that are conserved throughout eukaryotes (1, 2). DAG that is generated by PA dephosphorylation can be acylated to form TAG, an important source of fatty acids and stored energy that is deposited in specialized organelles, lipid droplets (3). PA-derived DAG can also be used for the synthesis of the membrane phospholipids phosphatidylethanolamine (PE) and phosphatidylcholine (PC), through the Kennedy pathway (4). Lipins can also influence PA and DAG pools involved in diverse signaling cascades (5, 6). In addition to their function as lipid metabolic enzymes, lipins contain nuclear localization signals and can translocate in the nucleus of hepatocytes and adipocytes where they regulate gene expression by modulating the activity of key transcription factors such as PPARγ coactivator 1/PPARα, PPARγ, or sterol regulatory element-binding protein 1 (7–11). Therefore, lipins can influence cell physiology at multiple levels. Besides lipins, eukaryotes posses a second class of PAP enzymes, known as lipid phosphate phosphatases (or PAP2 enzymes) (12). Unlike lipins, lipid phosphate phosphatases do not require Mg^2+^ for their activity and do contain multiple transmembrane domains, and their catalytic site faces the extracellular/lumenal face of membranes.

Although organisms such as fungi, flies, and worms express only one lipin, mammals express three: lipins 1, 2, and 3 (2, 13). The three lipin paralogues display distinct but overlapping patterns of expression in mouse and human tissues. Lipin 1 is more highly expressed in adipose tissue and skeletal muscle, lipin 2 is more highly expressed in liver and brain, and lipin 3 is more highly expressed in tissues of the gastrointestinal tract (2). When compared in mouse cell extracts containing overexpressed lipins, the specific PAP activity of lipin 1 is significantly higher than those of lipin 2 or 3 (2). Lipin 1 exists in three isoforms that are generated by alternative splicing: lipin 1α, 1β, and 1γ (14, 15). In most cell types, lipin 1 partitions between the cytoplasm and the nucleus, with the 1β isoform predominantly cytoplasmic and the 1α isoform mostly nuclear (14, 16–18). Lipin 1γ has been localized to lipid droplets (15). Comparison of the activities of the three recombinant lipin 1 isoforms shows that their specific PAP activity is in the order of 1α > 1β > 1γ (19).

Deleterious mutations in the lipin 1 gene in the fatty liver dystrophy (*fld*) mouse, result in a lipodystrophic phenotype characterized by severe deficiency in adipose tissue mass and peripheral neuropathy (13). Recently, a rat model has also linked lipin 1 function to adipose tissue homeostasis (20). This phenotype is consistent with the requirement of lipins for DAG production and the lack of TAG stores in the *fld* mouse. However, loss of lipin 1 also inhibits adipogenesis at an early stage that precedes the accumulation of TAG (21, 22), therefore making it impossible to assess the specific roles of lipin 1 in lipid and membrane homeostasis at later stages of differentiation. In this study, we examine, for the first time, the requirement of lipins in these processes after initiation of adipogenesis of 3T3-L1 cells but before the formation of TAG-filled lipid droplets. We found that depletion of lipin 1 at day 4 of differentiation results in an increase of lipin 2, whereas lipin 3 protein levels were undetectable in 3T3-L1 or mouse fractionated fat extracts. We therefore characterized the effects of the combined lipin 1 and 2 depletion after day 4 of adipogenesis. We find that this results in a loss of PAP activity and an increase of PA levels, although adipocytes still accumulate TAG after the down-regulation of lipins. We showed that the combined loss of lipin 1 and 2 causes a striking fragmentation of lipid droplets but, surprisingly, no significant changes in total lipid droplet volume. This is due to loss of the PAP activity of lipin 1, whereas depletion of lipin 2 caused an increase of lipid droplet volume per cell. We propose that in addition to their function during early adipogenesis, lipins are also implicated in lipid droplet biogenesis and maintenance at a later stage of adipocyte differentiation.

## EXPERIMENTAL PROCEDURES

### 

#### 

##### Tissue Culture

3T3-L1 preadipocytes were cultured and differentiated as described previously (23). Briefly, cells were seeded at 2 × 10^5^ cells in a well of a 6-well plate and cultured in DMEM (E15-011; PAA Laboratories) with 20 mm of l-glutamine (M11-004; PAA Laboratories), 1 unit/ml of penicillin/streptomycin (P11-010; PAA Laboratories), 10% of newborn calf serum (N4637; Sigma). Three days after confluency, the cells were grown in DMEM with 20 mm of l-glutamine, 1 unit/ml of penicillin/streptomycin, 10% of fetal bovine serum (Hyclone), 1 μm of insulin (Actrapid; Novo Nordisk), 0.5 mm of 3-isobutyl-1-methilxanthine (I7018; Sigma), and 1 μm of dexamethasone (D4902; Sigma) for 2 days to induce differentiation. Then the culture media were changed to DMEM with 1 μm of insulin on day 2 and DMEM with 20 mm of l-glutamine, 1 unit/ml of penicillin/streptomycin, and 10% of newborn calf serum every 2 days on afterward.

##### Plasmids

The shRNA lipin 1, lipin 2, and control (luciferase) vectors used were described previously (24). To construct the pLXIN-Lpin3-HA, the Lpin3 gene was amplified from day 12 differentiated 3T3-L1 adipocytes and subcloned into a pLXIN vector with a single HA tag immediately prior the stop codon. The sequences of the primers used (mL3–111F, mL3 + 2682R, mL3Xho5, and mL3HANotI) are listed in Table 1. Human lipin 1β was amplified from HeLa M cDNA and subcloned in a pLXIN vector with a C-terminal GFP tag. The catalytically dead human lipin 1β PAP mutant (D714E) was generated by PCR-mediated mutagenesis.

**TABLE 1:**
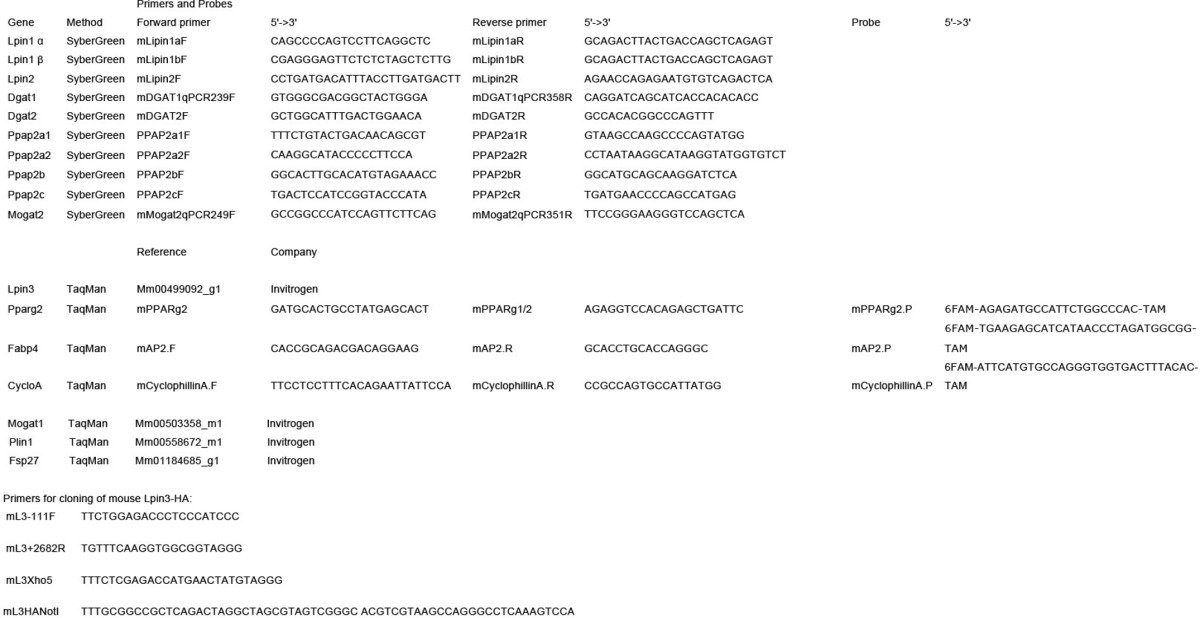
**Oligonucleotide primers used in this study**

##### Flow Cytometry Analysis

3T3-L1 adipocytes were grown in 6-well plates and collected by trypsin-EDTA release. The cell suspension was centrifuged at 800 × *g*, and the cell pellet was stained by HCS LipidTOX^TM^ Deep Red neutral lipid stain (Invitrogen) for 30 min in the CO_2_ incubator. The samples were then passed through a 70-μm Nylon cell strainer (BD Falcon) into a tube prior to flow cytometric determination of lipid droplet labeling using a Becton Dickinson FACSCalibur flow cytometer. Lipid droplet labeling was measured by using the FLA-1 and 4 lasers, and the flow cytometer data were analyzed with FlowJo software.

##### Adipose Tissue Fractionation

Epididymal fat pads were obtained from three C57BL/6 male mice, rapidly washed in warm PBS, and finely diced into small pieces (10–30 mg). A small piece was frozen in dry ice (total adipose tissue). The remaining tissue was incubated in medium 199 (22350-029; Invitrogen) plus 4% BSA and 2 mg/ml of collagenase type I (C6885; Sigma) for 40 min under agitation at 37 °C. Cells were separated by filtration through a 100-μm pore Nylon cell strainer (352360; BD Falcon). Media that passed the mess were transferred into one 15-ml tube and centrifuged at 400 × *g* for 5 min. The pellet, containing the stromovascular fraction (SVF), and the supernatant, containing the mature adipocytes, were separated. The mature adipocyte fraction was harvested carefully, washed thrice with 4 volumes of PBS, immediately frozen in dry ice, and stored at −80 °C. The SVF pellet was washed twice and incubated with 0.5 ml of fresh erythrocyte lysis buffer (10 mm KHCO_3_, 150 mm NH_4_Cl, EDTA 0,1 mm) for 5 min at room temperature. The pellet was washed twice more in PBS and immediately frozen.

##### RNAi Methods

Duplex siRNAs used in this study were control nontargeting (Nt1; Dharmacon catalog no. D-001810-01), mouse Lpin1 (mL1#1; Ambion catalog no. s66131), mouse Lpin1 (mL1#10; Dharmacon catalog no. J-040913-10), mouse Lpin2 (mL2#9; Dharmacon catalog no. J-059435-09), and mouse Lpin2 (mL2#12; Dharmacon catalog no. J-059435-12). siRNAs were dissolved in nuclease-free water (Qiagen) and used at 10 nm. In all knockdown experiments, 3T3-L1 cells grew in a 6-well plate and were transfected by these siRNAs on days 4 and 6 after induction of differentiation using 4 μl/well of Lipofectamine RNAiMAX (Invitrogen) in 400 μl/well of OptiMEM (Invitrogen).

##### Retroviral Infections

The Phoenix system was employed for retroviral infections (25). pSirenRetroQ or pLXIN constructs carrying the various shRNA or human lipin 1β-GFP constructs, respectively, were transfected to the Phoenix cell line by TransIT 293T transfection reagent (Mirus Bio) according to the manufacturer's instructions. Retroviral infectious media from the Phoenix cultures were used for infection to 3T3-L1 cells. Infected cell lines were selected by 1 μg/ml puromycin for pSirenRetroQ or 400 μg/ml of G418 for pLXIN expression.

##### Quantitative PCR Analysis

Total RNA was isolated using the QIAshredder (Qiagen) and RNeasy kit (Qiagen) following the manufacturer's protocol. cDNA was synthesized using the high capacity cDNA reverse transcription kit (Applied Biosystems) following the manufacturer's instructions. Quantitative real time PCR was performed as described previously (24). The primers and probes used are described in Table 1. All reverse transcription-PCRs were quantified using the ΔΔ*C*_t_ method and cyclophilin A as an internal normalizing reference gene.

##### Cell Extracts, Western Blot Analysis, and Antibodies

Cell extracts were performed in ice-cold PBS with 0.2% SDS and Complete, EDTA-free protease inhibitor mixture (Roche Applied Science). Lysates were resolved by SDS-PAGE, transferred to nitrocellulose membranes (Whatman), blocked in 5% nonfat dry milk in PBS containing 0.01% Tween 20 for 1 h at room temperature, and incubated with the primary antibody overnight at 4 °C and with the secondary antibody for 1 h. The Western blots were developed with ECL (GE Healthcare). Antibodies against the following proteins were used: lipin 1 and 2 (24), FABP4 (sc-18661; Santa Cruz Biotechnology), PPARγ (2443; Cell Signaling), perilipin 1 (4854; VALA Sciences), Fsp27 (26), actin (A2066; Sigma), and HA (sc-805; Santa Cruz Biotechnology). HRP-conjugated secondary antibodies against rabbit IgG (20321-200; Alpha Diagnostic) and against goat IgG (A8919; Sigma) were used for Western analysis. To generate antiserum against lipin 3, the nucleotide sequence of human lipin 3, corresponding to amino acid residues 155–365, were cloned into pGEX4T1, and the GST-tagged fusion protein was affinity-purified from bacterial lysate supernatants using glutathione-Sepharose 4B (Amersham Biosciences, GE Healthcare). Antibodies against the GST-lipin 3 fusion were raised in rabbits by four subcutaneous injections over 3 months.

##### TAG Quantification

3T3-L1 cells in 6-well plates were lysed with 150 μl of 5% Triton X-100/plate, then processed by slowly warming up to 95 °C, vortexed for 2 s, and held at 95 °C for a further 5 min before slowly cooling to room temperature. This was repeated once more before the samples were centrifuged for 5 min at 13,000 × *g*, and 100 μl of the supernatant was collected. A sample volume of 20 μl was then loaded onto a triglyceride flex reagent cartridge (Dade Behring), and triglyceride concentrations were assayed using a lipase reaction at 37 °C on a Dade Behring Dimension RXL analyzer.

##### PA Quantification

Lipids from 3T3-L1 adipocytes at day 8 after induction of differentiation were extracted, and cellular PA levels were quantified with an enzymatic assay (27) using the total phosphatidic acid assay kit (Cayman Chemical Co.) following the manufacturer's instructions.

##### Lipid Extraction and Liquid Chromatography-Mass Spectrometry

Lipids were extracted from cells by the method of Bligh and Dyer (28) with minor modifications (29). The lipids were analyzed in a single chromatographic method (29) using a Dionex UltiMate 3000 LC system coupled to an Applied Biosystems 4000 Q Trap mass spectrometer with an electrospray ionization source. The high performance liquid chromatography column was a Waters SpherisorbS5W 4.6 × 100-mm silica cartridge, 5-μm particle size, with a Waters Spherisorb S5W 4.6 × 10-mm guard cartridge. TAG amounts were adjusted for the internal standard and estimated from a standard curve containing seven TAG standards (triolein, tritridecanoic acid, tripalmitolein, trilinolein, tripalmitin, tristearin, and trimyristin). The standard curve mixture used for quantitation of phospholipids included at least one compound from each class. The compounds used were dioleoyl-PC, dimyristoyl-PC, dieicosenoyl-PC, dioleoyl-PE, dioleoyl-phosphatidylinositol (dioleoyl-PI), dioleoyl-phosphatidylserine (dioleoyl-PS), and dioleoyl-PA. For PI, a mixture extracted from liver was used for the standard curve.

##### PAP Activity Assays

3T3-L1 cells were lysed by sonication in 300 μl of 50 mm Tris-HCl, pH 7.5 buffer containing 0.25 m sucrose, 10 mm 2-mercaptoethanol, and protease inhibitors (1 mm benzamidine, 0.5 mm PMSF, 5 μg/ml of aprotinin, leupeptin, and pepstatin). The cell lysates were centrifuged at 1,000 × *g* for 10 min at 4 °C, and the supernatant was used for the measurement of PAP activity. PAP activity was measured by following the release of water-soluble ^32^P_i_ from chloroform-soluble [^32^P]PA at 37 °C as described previously (19, 30). The radioactive substrate was synthesized enzymatically from dioleoyl-DAG and [γ-^32^P]ATP with DAG kinase (30). The reaction mixture for total PAP activity (Mg^2+^-dependent and Mg^2+^-independent) contained 50 mm Tris-HCl, pH 7.5 buffer, 0.5 mm MgCl_2_, 10 mm 2-mercaptoethanol, 1 mm dioleoyl [^32^P]PA (10,000 cpm/nmol), 10 mm Triton X-100, and an appropriate amount of enzyme protein in a total volume of 0.1 ml. Mg^2+^-independent (*e.g.*, PAP2) activity was measured under the same reaction conditions except for the substitution of 1 mm EDTA for 0.5 mm MgCl_2_. The Mg^2+^-dependent activity (*e.g.*, PAP1) was calculated by subtracting the Mg^2+^-independent activity from the total PAP activity (5, 24). Enzyme assays were conducted in triplicate, and the average S.D. was ± 5%. All enzyme reactions were linear with time and protein concentration. A unit of enzymatic activity was defined as the amount of enzyme that catalyzed the formation of 1 nmol of product per minute.

##### Imaging and Three-dimensional Analysis

3T3-L1 cells were grown on poly-d-lysine-coated glass coverslips until confluent and then differentiated as described above. Lipid droplets were stained by 10 μg/ml of BODIPY 493/503 (D-3922; Invitrogen) or HCS LipidTox Deep Red neutral lipid stain (H34477; Invitrogen) for 30 min in CO_2_ incubator at 37 °C before fixation with 4% paraformaldehyde. For lipin 1 immunofluorescent labeling, 3T3-L1 cells were permeabilized by 0.2% saponin in PBS for 5 min at room temperature and washed in PBS with 3% BSA (PBSB) three times. To confirm the knockdown, purified anti-Lipin1 antibody was used for staining the cells as 1/1000 dilution in PBSB and incubated for 1 h at room temperature. After washing the cells in PBSB three times, Cy5-conjugated anti-rabbit IgG was used as secondary antibody to label anti-Lipin 1 antibody. Coverslips were then mounted onto glass slides using ProLong Gold antifade reagent (Invitrogen). Confocal microscopy was performed using LSM 710 confocal microscopy (Zeiss), and three-dimensional images were reconstructed from 15 slices of each cell by Zen software (Zeiss). Three-dimensional images were imported to Volocity three-dimensional image analysis software (PerkinElmer Life Science) to measure size and number of lipid droplets in each cell. The software was run with optimized parameters of find object, remove noise from object, separate touching objects, and exclude object size to determine each lipid droplet in cells, which were selected manually.

## RESULTS

### 

#### 

##### Lipin 1, 2, and 3 Protein Expression Levels in Mouse Adipocytes

**FIGURE 1.**
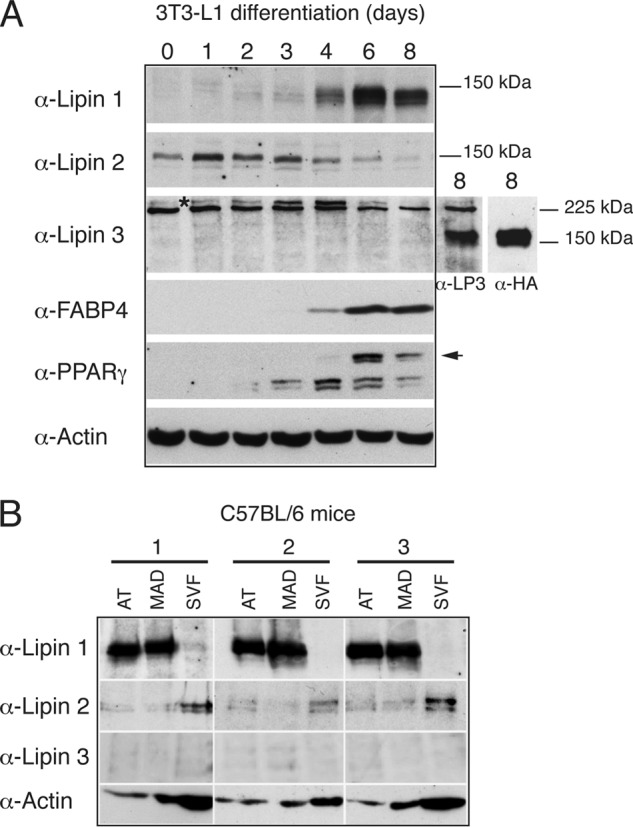
**Lipin 1, 2, and 3 protein expression patterns in mouse adipocytes.** *A*, 3T3-L1 cells were induced to differentiate at day 0 by addition of an insulin-containing mixture as described under “Experimental Procedures.” Cell extracts were prepared at the indicated time points, and 5 μg of each sample was analyzed by Western blot with the indicated antibodies. The *asterisk* indicates the protein band recognized by the lipin 3 antibody. The *arrow* points to the band corresponding to PPARγ2. The two additional lipin 3 lanes represent extracts from 3T3-L1 cells retrovirally infected with a pLXIN-Lpin3-HA(1X) fusion (5 μg each) and analyzed with anti-Lipin 3 or anti-HA antibodies, respectively. Molecular mass markers (kDa) are indicated. *B*, lipin 1, 2, and 3 expression in fractionated mouse adipose tissue. Epididymal fat from three male C57BL/6 mice was fractionated as described under “Experimental Procedures.” 10 μg of each fraction was resolved by SDS-PAGE followed by Western blotting with the indicated antibodies. *AT*, adipose tissue; *MAD*, mature adipocytes.

To address their roles after initiation of the adipogenic program, we sought first to establish the protein expression pattern of the three lipins in mouse adipocytes. We have previously shown that lipins 1 and 2 displayed a reciprocal pattern of expression during 3T3-L1 adipogenesis (Ref. 24 and Fig. 1*A*). However, there is no information on the protein levels of lipin 3 in 3T3-L1 cells. When probing 3T3-L1 extracts with our lipin 3 antibody, two closely migrating protein bands at or above the 225-kDa protein marker were identified (Fig. 1*A*). This is more than double the predicted mass of lipin 3. To address whether these bands correspond to lipin 3, we cloned from 3T3-L1 cells the Lpin3 cDNA, fused it to a single HA tag and expressed the Lipin3-HA fusion from a retroviral vector in 3T3-L1 cells. In these cell extracts, both the lipin 3 and HA antibodies recognized the same one band, close to 150 kDa, which is also the apparent mobility of lipins 1 and 2 (Fig. 1*A*), suggesting that the 225-kDa band reacted nonspecifically with anti-lipin 3 antibodies. We conclude that the lipin3 antibody can detect overexpressed mouse lipin 3 in adipocyte extracts but not at endogenous levels, suggesting that, if present, lipin 3 protein is expressed at low amounts. Consistent with this, we found that Lpin3 mRNA is present at very low levels in 3T3-L1 cells (data not shown). We next assessed the expression of lipins in adipocytes isolated from mouse fat. Epidydimal adipose tissue from three mice was fractionated to separate mature adipocytes from the other cell types present in the adipose tissue (SVF). Lipin 1 was strongly enriched in mature adipocytes but absent from SVF cells, whereas lipin 2 was present in the SVF but could be also detected in mature adipocytes (Fig. 1*B*). We could not detect endogenous lipin 3 in either adipocytes or SVF cells. Thus 3T3-L1 and mouse adipocytes show a comparable pattern of lipin expression, with lipin 1 strongly expressed in mature adipocytes, whereas lipin 2 is expressed at lower levels when compared with the SVF cells. We therefore decided to focus on lipins 1 and 2.

##### Opposing Roles of Lipins 1 and 2 in Adipogenesis

**FIGURE 2.**
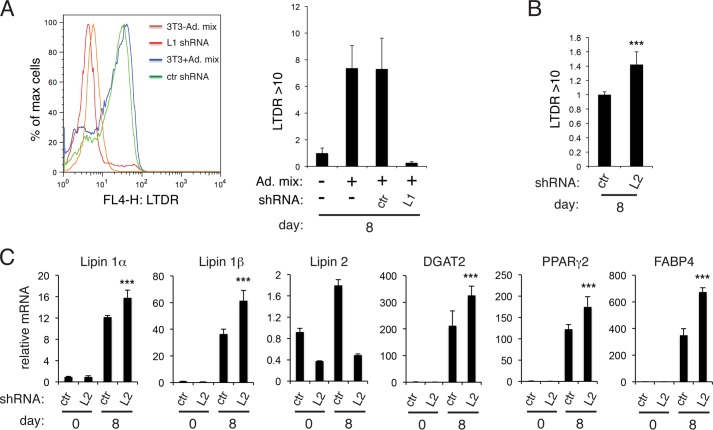
**Distinct effects of lipin 1 and lipin 2 depletion in 3T3-L1 preadipocytes.** *A*, FACS-based quantification of neutral lipid labeling in untransfected 3T3-L1 (3T3-Ad. mix), untransfected 3T3-L1 following induction of adipogenesis (3T3 + *Ad. mix*), or transfected with control (*ctr*) or lipin 1 (*L1*) shRNA and induction of adipogenesis. *Left panel*, fluorescence of the cells labeled with LTDR at day 8. *Right panel*, quantification of LTDR > 10 shown in the *left panel*. The values are normalized to untransfected 3T3-L1 at day 8 and are the means of four experiments ± S.D. *B*, 3T3-L1 cells were transfected with retroviral vectors expressing control shRNA (*ctr*) or lipin 2 shRNA (L2), induced to differentiate, labeled with LTDR at day 8, and assayed as in *A*. The values are normalized to control shRNA. The values represent means of four experiments ± S.D. ***, *p* < 0.005 for the comparison with control. *C*, qPCR was performed to quantify expression of the indicated genes in 3T3-L1 cells from *B* at days 0 or 8 after induction of adipogenesis. The data are normalized to cyclophilin A mRNA and to control at day 0. The values are means of two independent experiments, and within each experiment shRNA transfections were performed in triplicate. ***, *p* < 0.005 for the comparison with control.

Next, we compared the effects of lipin depletion before and after initiation of adipogenesis. As expected, retroviral shRNA-mediated knockdown of lipin 1 prior to differentiation of 3T3-L1 cells resulted in an almost complete loss of total neutral lipid, quantified by FACS analysis of cells labeled with LipidTox Deep Red (LTDR) (Fig. 2*A*), TAG, and PPARγ2 induction (data not shown). Interestingly, retroviral infection of a lipin 2 shRNA construct in preadipocytes had the opposite effect: 3T3-L1 cells accumulated more neutral lipid at day 8 (Fig. 2*B*), and this was accompanied by a significant increase in lipin 1β, the more highly induced PAP enzyme in mature adipocytes (14), the acyl-CoA:diacylglycerol acyltransferase DGAT2, the mature adipocyte marker FABP4, and PPARγ2 (Fig. 2*C*).

##### Combined Down-regulation of Lipins 1 and 2 after Initiation of Adipocyte Differentiation

**FIGURE 3.**
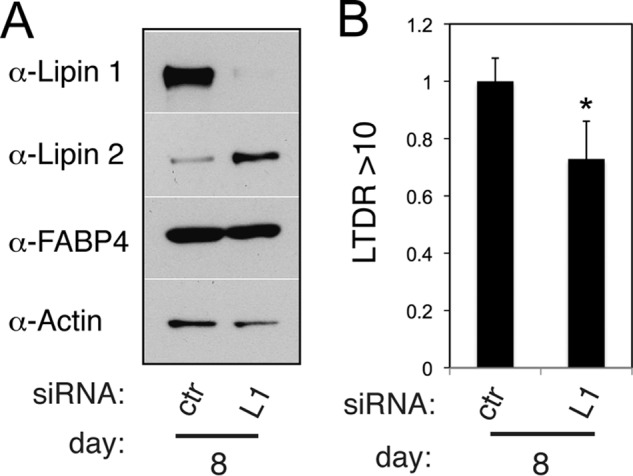
**siRNA-mediated down-regulation of lipin 1 after initiation of adipogenesis of 3T3-L1 cells.** *A*, 3T3-L1 adipocytes were transfected with nontargeting (control, *ctr*) or lipin 1 (*L1*) siRNA at days 4 and 6 after induction of differentiation. Cell extracts were prepared at day 8, and 5 μg of each sample was analyzed by Western blot with the indicated antibodies. *B*, cells from *A* were labeled with LTDR and assayed as in Fig. 2*B* at day 8. The values are means ± S.D. of four experiments and normalized to control siRNA. *, *p* < 0.05 for the comparison with control.

**FIGURE 4.**
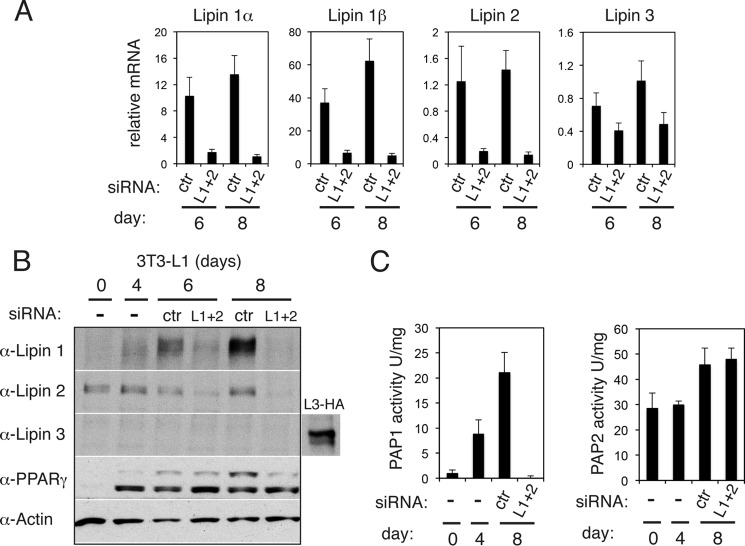
**Combined down-regulation of lipins 1 and 2 in 3T3-L1 cells.** *A*, 3T3-L1 adipocytes were transfected with nontargeting (control, *ctr*) or both lipin 1 and lipin 2 (*L1* + *2*) siRNAs at days 4 and 6 after induction of differentiation. qPCR analysis was performed at the indicated days to quantify lipin 1α, 1β, 2, and 3 mRNA levels. The data are normalized to cyclophilin A mRNA and to control at day 1. The values are means ± S.D. of three independent experiments, and within each experiment siRNA transfections were performed in triplicate. *B*, extracts from cells in *A* were prepared at the indicated time points, and 5 μg of each sample was analyzed by Western blot with the specified antibodies. The blot shown is representative of six experiments. *C*, PAP1 and PAP2 activities were measured in 3T3-L1 cell extracts transfected with nontargeting (control, *ctr*) or lipin 1 and lipin 2 (*L1* + *2*) siRNAs as described under “Experimental Procedures.” The values are means ± S.D. of four experiments.

To assess the roles of lipin 1 after the initiation of adipogenesis, we set up a siRNA transfection protocol to down-regulate lipin 1 expression at days 4 and 6 of differentiation before assaying its function at day 8, when control 3T3-L1 adipocytes are filled by large lipid droplets. We chose day 4 as the initial time point for the lipin 1 knockdown, because this is the latest point after the start of adipogenesis when cells are still devoid of medium or large sized droplets. Transfection of nontargeting siRNAs were applied to control adipocytes at the same time points. Surprisingly, when lipin 1 was knocked down at this time point, there was only a minor change in neutral lipid levels, and cells expressed comparable levels of FABP4 at day 8 as control cells (Fig. 3). However, we noticed that the protein levels of lipin 2 were significantly elevated when lipin 1 was knocked down at this stage of differentiation (Fig. 3*A*). Although up-regulation of lipin 2 cannot compensate for the loss of lipin 1 during initiation of 3T3-L1 adipogenesis (24), it is not known to what extent lipin 2 can perform such a role at later stages, when cells form lipid droplets and accumulate TAG. To rule out the possibility of compensatory effects that could mask some of the phenotypes of lipin 1 knockdown, we performed a combined transfection of both lipin 1 and 2 siRNAs at day 4, followed by a second transfection of both lipin siRNAs at day 6 of differentiation. Applying this protocol, mRNA levels of lipin 1α, lipin 1β, and lipin 2 were efficiently down-regulated (Fig. 4*A*). Lipin 3 mRNA could be detected in control cells transfected with the nontargeting siRNAs at very low levels, as judged by the observed *C*_t_ values, which decreased further in the double lipin1/2 knockdown cells (Fig. 4*A*). The basis of this change of lipin 3 mRNA is currently not clear. However, at the protein level, lipin 3 was undetectable in control cells, and no change was observed in the double lipin 1 and 2 knockdown cells (Fig. 4*B*). To validate the effective down-regulation of lipin 1 and 2 function, we assayed PAP activity in adipocyte extracts following the transfection of the lipin 1 and 2 siRNA oligonucleotides. Consistent with previous data (24), Mg^2+^-dependent PAP activity (PAP1), which is lipin-dependent, increased during differentiation of control adipocytes (Fig. 4*C*). Importantly, this activity was almost undetectable in the lipin 1/2 knockdown adipocytes (Fig. 4*C*), whereas the Mg^2+^-independent (PAP2) activity did not change. Taken together, these data showed that the protein and activity levels of lipins 1 and 2 are efficiently down-regulated in 3T3-L1 adipocytes when their expression was reduced after day 4 of differentiation.

##### Effects of Lipin 1/2 Knockdown on the Expression of Genes Involved in Adipocyte Function and Lipid Composition

**FIGURE 5.**
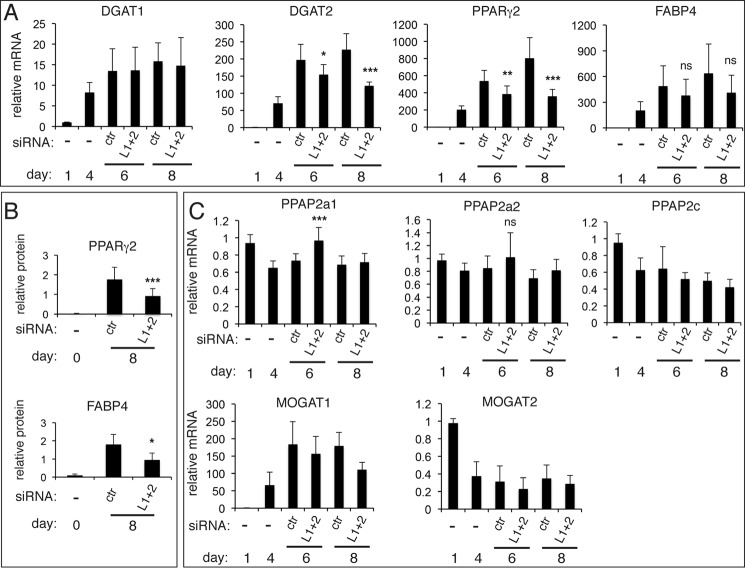
**Effects of combined lipin 1 and lipin 2 down-regulation on the mRNA and protein levels of adipogenic and lipogenic markers.** *A*, 3T3-L1 adipocytes were transfected with nontargeting (control, *ctr*) or lipin 1 and lipin 2 (*L1* + *2*) siRNAs at days 4 and 6 after induction of differentiation. qPCR analysis was performed at the indicated days to quantify the DGAT1, DGAT2, PPARγ2, and FABP4 gene expression during differentiation. The data are normalized to cyclophilin A mRNA and to control at day 1. The values are means ± S.D. of three independent experiments, and within each experiment siRNA transfections were performed in triplicate. *B*, quantification of protein levels of PPARγ2 and FABP4. Extracts from cells in *A* were analyzed by Western blot using anti-PPARγ and anti-FABP4 antibodies, the corresponding bands were scanned, and intensities were normalized to actin levels from the same protein extract. The values represent means from four experiments ± S.D. *, *p* < 0.05; **, *p* < 0.01; ***, *p* < 0.005 for the comparison with control. *C*, qPCR was performed as in *A* to quantify the indicated genes. ***, *p* < 0.005 for the comparison with control. *ns*, not significant when compared with control.

We examined the expression of genes required for adipocyte function and maintenance in lipin 1/2 knockdown 3T3-L1 cells. mRNA levels of PPARγ2 were reduced (Fig. 5*A*). DGAT1 mRNA levels were not affected, whereas DGAT2 mRNA, the more highly induced paralogue during adipocyte differentiation, decreased by 50% compared with the nontargeting siRNA (Fig. 5*A*). Protein levels of both PPARγ2 and FABP4 showed similar reductions—46 and 31% decrease, respectively—when compared with the nontargeting siRNA-transfected cells (Fig. 5*B*).

**FIGURE 6.**
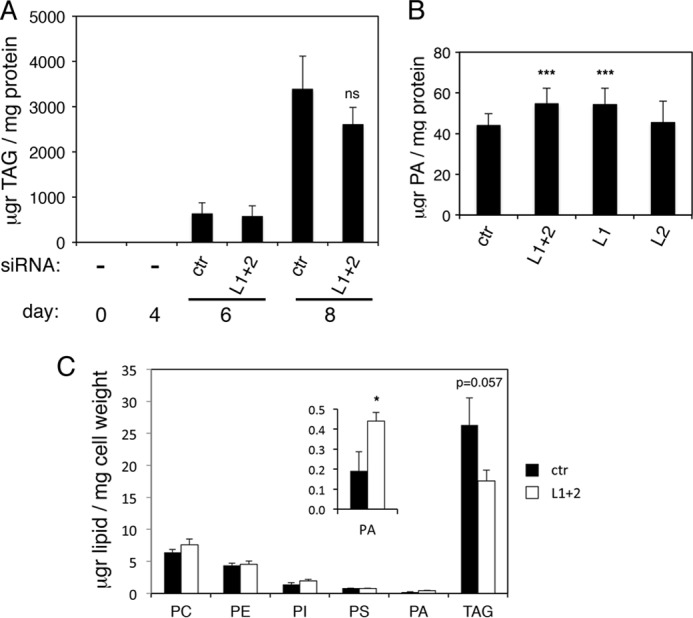
**Effects of combined lipins 1 and 2 down-regulation on the masses of lipids in 3T3-L1 adipocytes.** *A*, 3T3-L1 adipocytes were transfected with nontargeting (control, *ctr*) or lipin 1 and lipin 2 (*L1* + *2*) siRNAs at days 4 and 6 after induction of differentiation. Cells from days 0, 4, 6, and 8 were collected, and total TAG was determined as described under “Experimental Procedures.” The values are means ± S.D. of four experiments. *ns*, not significant for the comparison with ctr (*p* = 0.091). *B*, 3T3-L1 adipocytes were transfected with nontargeting (control, *ctr*) or lipin 1 and lipin 2 (*L1* + *2*), lipin 1 only (*L1*), or lipin 2 only (*L2*) siRNAs as in *A*. Total PA levels were calculated enzymatically as described under “Experimental Procedures.” The values are means ± S.D. of seven experiments. ***, *p* < 0.005 for the comparison with control. *C*, 3T3-L1 adipocytes were differentiated and transfected with siRNAs as in *A*. Lipid extracts were prepared from day 8 adipocytes as described under “Experimental Procedures,” and the amounts of TAG and major phospholipid classes were analyzed by high performance liquid chromatography-mass spectroscopy. The values are means ± S.D. of two experiments. *, *p* < 0.05 for the comparison with control.

Taken together, these data suggest that adipocyte maturation is impaired when lipins 1 and 2 are down-regulated at day 4 of differentiation. Despite these defects, FACS-based quantification showed that the lipin1/2 knockdowns accumulate neutral lipid during differentiation (data not shown). To investigate the lipid changes in these cells, we performed a time course of TAG buildup during differentiation with or without lipin 1 and 2 down-regulation. We found that mature adipocytes could still accumulate TAG after day 4, when lipins 1 and 2 were first down-regulated, although there was a modest, but not significant, decrease when compared with the TAG levels of control adipocytes transfected with the nontargeting siRNA (L1 + 2 cells: 77% of control TAG, *p* = 0,091; Fig. 6*A*). Analysis of total cellular PA levels using an enzymatic assay showed that lipin1/2 knockdown cells contained more PA than control adipocytes, consistent with the loss of PAP activity, and that this difference was due to the depletion of lipin 1 (Fig. 6*B*). To further examine in more detail how the lipin depletion affects the masses of TAG and phospholipids and their respective fatty acyl compositions, lipid extracts from either knockdown or control adipocytes, both at day 8, were analyzed by high performance liquid chromatography-mass spectroscopy. Surprisingly, the levels of the abundant structural phospholipid classes, PE and PC, did not show any major changes (Fig. 6*C*). PA mass levels exhibited an increase of 229% following lipin down-regulation. The depletion of lipins 1/2 did not cause any striking changes in the fatty acyl moieties of these phospholipids (data not shown). Consistent with the time course data above, the lipin 1/2 knockdown cells accumulated 67% of total day 8 TAG mass when compared with the control adipocytes (*p* = 0.057; Fig. 6*C*) and showed no significant differences in their fatty acyl chain composition (data not shown). These data raise the possibility that another pathway may be able to compensate and provide DAG for TAG synthesis in these cells. However, mRNA levels of PPAP2a1, PPAPa2, and PPAP2c, all encoding Mg^2+^-independent PAP enzymes, did not considerably increase following lipin depletion (Fig. 5*C*, with the exception of a 30% increase of PPAP2a1 at day 6), consistent with the PAP2 activity data (Fig. 4*C*). Similarly, mRNA levels of MOGAT1 and 2, belonging to the monoacylglycerol acyltransferase family involved in TAG production in enterocytes, did not increase (Fig. 5*C*). Alternative mechanisms may thus provide TAG in the lipin-depleted adipocytes (see “Discussion”).

##### Depletion of Lipins 1 or 2 after the Initiation of Adipogenesis Has Different Effects on Lipid Droplet Size and Number in 3T3-L1 Adipocytes

**FIGURE 7.**
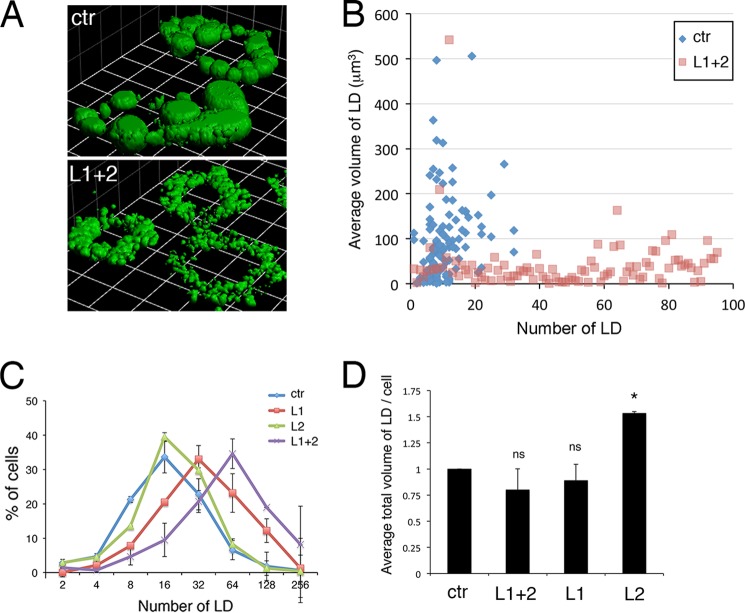
**Opposing effects of lipin 1 and lipin 2 depletion in lipid droplet biogenesis of 3T3-L1 cells.** *A*, 3T3-L1 adipocytes were transfected with nontargeting (control, *ctr*) or lipin 1 and lipin 2 (*L1* + *2*) siRNA at days 4 and 6 after induction of differentiation. Day 8 adipocytes were fixed, stained with BODIPY 493/503, and imaged with an LSM 710 confocal microscope. Three-dimensional reconstruction from Z-stacks to visualize lipid droplets was performed as described under “Experimental Procedures.” Representative three-dimensional reconstructions from control or L1 + 2 adipocytes are shown. Rectangle side size was 100 μm. *B*, scatter plot of lipid droplet number (*x axis*) *versus* average lipid droplet volume (*y* axis) per cell in control or L1 + 2 3T3-L1 day 8 cells. Representative data from three separate experiments are shown. *C*, distribution of lipid droplet numbers in 3T3-L1 day 8 cells transfected with nontargeting (control, *ctr*), lipin 1 and lipin 2 (*L1* + *2*), lipin 1 only (*L1*), or lipin 2 only (*L2*) siRNA at days 4 and 6. *D*, average total lipid droplet volumes per cell for the experiments shown in *C*. The values are normalized to the lipid droplet volumes per cell of the control (*ctr*) 3T3-L1 transfectants, set at 1. The experiments in *C* and *D* represent means ± S.D. of at least three (control and L1 + 2) or two (L1 and L2) experiments. The total number of cells analyzed: control, 423; L1 + 2, 334; L1 203; and L2, 235. *, *p* < 0.05 for the comparison with control. *ns*, not significant for the comparison with control (*p* = 0.2 for L1 + 2; *p* = 0.5 for L1). *LD*, lipid droplets.

Because lipin 1/2 knockdown cells still contain significant TAG at day 8 and recent evidence implicates the yeast lipin Pah1p in lipid droplet biogenesis (31), we examined droplet morphology in these cells using the lipophilic dye BODIPY 493/503. This revealed that double lipin knockdown cells exhibited a dramatic increase in the number of smaller lipid droplets (Fig. 7*A*). Differentiated adipocytes treated with the control siRNA contained between 4 and 20 droplets, whereas in lipin-depleted cells this number increased drastically with a concurrent decrease of individual droplet volume (Fig. 7, *B* and *C*). However, when quantified by three-dimensional image reconstruction of confocal Z-slices, the total lipid droplet volume per adipocyte did not change significantly in lipin-depleted cells (80% of the volume of control adipocytes, *p* = 0.2; Fig. 7*D*). To examine whether droplet fragmentation was due to an additive effect of depleting the two lipins, we down-regulated individually either lipin 1 or lipin 2 at days 4 and 6 and quantified droplet numbers and total volume at day 8. We found that depletion of lipin 1 caused a similar defect as the double knockdown, whereas depletion of lipin 2 resulted in an increase of total droplet volume per cell without significantly affecting droplet numbers (Fig. 7*D*). Moreover, combining the two lipin siRNAs did cause a modest but reproducible increase of droplet numbers when compared with the single lipin 1 knockdown cells (Fig. 7*C*). Similar results in lipid droplet volume and numbers were obtained when both single and double lipin 1 and/or 2 knockdowns were performed with a different combination of siRNA oligonucleotides (data not shown).

**FIGURE 8.**
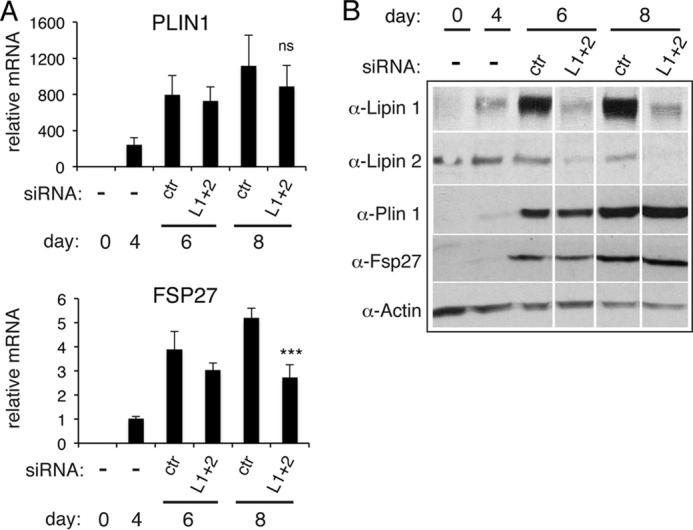
**Effects of combined lipin 1 and 2 down-regulation at days 4 and 6 on the mRNA and protein levels of Plin1 and Fsp27.** *A*, 3T3-L1 were transfected with nontargeting (control, *ctr*) or lipin 1 and lipin 2 (*L1* + *2*) siRNAs at days 4 and 6 after induction of differentiation. qPCR analysis was performed at the indicated time points to quantify Plin1 and Fsp27 mRNA levels. The data are normalized to cyclophilin A mRNA and to control at day 0 for Plin1 or day 4 for Fsp27. The values are means ± S.D. of three independent experiments, and within each experiment, siRNA transfections were performed in triplicate. *ns*, not significant for the comparison with control. ***, *p* < 0.001 for the comparison with control. *B*, extracts from cells in *A* were prepared at the same time points of differentiation, and 8 μg of each sample was analyzed by Western blot with the specified antibodies. The blot shown is representative of two experiments.

We asked whether lipin depletion could affect the expression of perilipin 1 (Plin1), the major adipocyte protein that coats lipid droplets (32), and Fsp27, whose mutation results in small and multilocular droplets, similar to the ones seen in lipin 1 knockdown cells (26, 33). Plin 1 mRNA did not significantly change, whereas Fsp27 mRNA did decrease in the lipin 1/2-depleted cells (Fig. 8*A*). However, levels of both proteins were not affected at day 6 or 8 of differentiation (Fig. 8*B*), suggesting that the defects in the lipin-depleted cells are not due to decreased stability of Plin 1 or Fsp27.

##### The PAP Activity of Lipin 1 Is Essential for Normal Lipid Droplet Size and Number in 3T3-L1 Adipocytes

**FIGURE 9.**
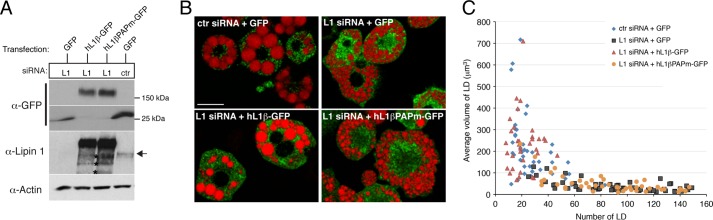
**The PAP activity of lipin 1 is essential for normal lipid droplet size and number in 3T3-L1 adipocytes.** *A*, 3T3-L1 preadipocytes were transfected with retroviral vectors expressing GFP, wild-type human lipin 1β-GFP (hL1β-GFP), or the catalytically dead human lipin 1β-GFP (hL1βPAPm-GFP). Following selection of stable transfectants, cells were induced to differentiate and transfected with nontargeting (control, *ctr*) or lipin 1 (*L1*) siRNAs at days 4 and 6 as described before. Cell extracts were prepared at day 8 of differentiation, and 1.5 μg of each sample was analyzed by Western blot with the specified antibodies. Two different parts of the α-GFP blot are shown corresponding to the full-length lipin 1β-GFP constructs and the GFP fragment alone. The *arrow* points to the endogenous lipin 1 protein band, and *asterisks* indicate the breakdown products of the human lipin 1β-GFP fusions. *B*, day 8 adipocytes from *A* were fixed, stained with LTDR, and imaged with an LSM 710 confocal microscope. *Red*, lipid droplets; *green*, GFP fusions. *Bar*, 30 μm. *C*, scatter plot of lipid droplet number (*x axis*) *versus* average lipid droplet volume (*y* axis) per cell from the indicated adipocytes. Quantification and analysis were performed as in Fig. 7*B*.

Lipin 1 has dual roles as PAP enzyme and transcriptional regulator of adipogenic genes during differentiation. Therefore we asked whether its PAP activity is required for rescuing the decreased lipid droplet size of lipin 1-depleted adipocytes. To perform these rescue experiments, we took advantage of the fact that the sequence of the human and mouse lipin 1 genes are different at the site targeted by the lipin 1 siRNA oligonucleotides, rendering human lipin 1 constructs resistant to silencing (Fig. 9*A*). We transfected 3T3-L1 preadipocytes using retroviral vectors expressing GFP, human lipin 1β-GFP, or human lipin 1β-PAPm-GFP where its phosphoacceptor site, DIDGT, has been mutated to EIDGT, resulting in a catalytically inactive enzyme. These cells were then differentiated and transfected with either lipin 1 or control nontargeting siRNAs at days 4 and 6, before imaging their lipid droplets at day 8 as described above. Expression of either of the two lipin 1β constructs did not significantly affect droplet biogenesis in control siRNA cells (data not shown). In the lipin 1 siRNA-treated cells, wild-type lipin 1β restored droplet size and number to levels comparable to those seen in control GFP cells, whereas lipin 1β-PAPm did not rescue the phenotype and displayed a droplet distribution similar to the lipin 1 knockdown cells expressing GFP (Fig. 9, *B* and *C*). These results indicate that the catalytic activity of lipin 1 is essential for proper lipid droplet biogenesis in adipocytes.

##### Depletion of Lipin 2 after Initiation of Differentiation Does Not Affect Expression of Genes Involved in Adipocyte Function

**FIGURE 10.**
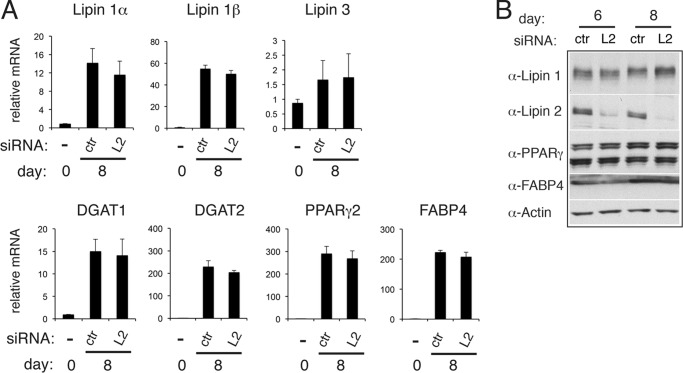
**Effects of lipin 2 down-regulation on the mRNA and protein levels of adipogenic and lipogenic markers.** *A*, 3T3-L1 were transfected with nontargeting (control, *ctr*) or lipin 2 (*L2*) siRNA at days 4 and 6 after induction of adipogenesis. qPCR analysis was performed at days 0 and 8 of differentiation to quantify the indicated genes. The data are normalized to cyclophilin A mRNA and to control at day 0. The values are means ± S.D. of two independent experiments, and within each experiment siRNA transfections were performed in triplicate. *B*, extracts from cells in *A* were prepared at days 6 and 8 of differentiation, and 5 μg of each sample was analyzed by Western blot with the specified antibodies. The blot shown is representative of three experiments.

Depletion of lipin 2 in preadipocytes resulted in increased lipid droplet labeling concomitant with a rise of adipogenic and lipogenic markers (Fig. 2, *B* and *C*). We asked whether this would be also the case when lipin 2 is depleted at days 4 and 6 of differentiation. As seen in Fig. 10, levels of lipin 1α and 1β, lipin 3, DGAT1, DGAT2, PPARγ2, and FABP4 mRNA, or PPARγ2 and FABP4 protein do not change in lipin 2-depleted cells. Therefore, lipin 2 deficiency at this stage of differentiation has no impact on the transcriptional regulation of adipocytes, whereas it still results in an increase in lipid droplet biogenesis.

## DISCUSSION

The presence of three lipins, sharing the same PAP activity and overlapping expression patterns in many mammalian tissues, raises two important questions: first, what are the functional differences between lipins 1, 2 and 3, in cell types where more than one lipin is active, and, second, to what extent does loss of one lipin paralogue affect the activity of the other(s)? Here we examined the roles of lipins after the initiation of differentiation of 3T3-L1 adipocytes. To bypass the essential function of lipin 1 at the early stages of adipogenesis and eliminate possible compensation by lipin 2 that was up-regulated in the absence of lipin 1, we sought to generate a setup where lipins are absent after the initiation of differentiation and evaluate their effect on TAG and lipid droplet formation.

Surprisingly, and unlike when lipin 1 is mutated or depleted in preadipocytes (8, 21, 22, 24), we found that following the combined lipin 1 and 2 siRNA transfection at days 4 and 6, cells did accumulate TAG at day 8. This could be due to another pathway that provides DAG for TAG synthesis. Lipin 3 was still present, but its protein levels were undetectable under the conditions of our experiments. Other studies have documented an increase in lipin 3 mRNA levels in various tissues when lipins 1 or 2 are mutated (2, 34, 35), but this was not observed in our experiments with the 3T3-L1 adipocytes. We therefore consider it unlikely that lipin 3 can support TAG synthesis in the lipin 1/2-depleted cells. We found no evidence for up-regulation of lipid phosphate phosphatase/PAP2 enzymes or MOGATs. Members of the sphingomyelin synthase or phospholipase C families may be involved, although it is unclear whether the levels or location of DAG generated by these enzymes would be appropriate for TAG production. An alternative possibility is that a residual PAP activity at the initial stages of the lipin knockdown may still be able to support TAG synthesis. A similar situation was described in hepatocytes from the *fld* lipin 1-deficient mouse following depletion of lipin 2 where cells with decreased PAP activity were still able to support TAG synthesis (34).

Our data are consistent with a role of lipins in lipid droplet biogenesis at a later stage of adipocyte differentiation. A recent study reported that specific acyltransferase paralogues of the *de novo* TAG biosynthetic pathway relocalize onto lipid droplets and are required for their expansion during fatty acid loading in fly and mammalian cells (36). Interestingly, in the absence of these enzymes, cells are depleted from large droplets (36). These data raise the possibility that lipin 1 may play a direct role in lipid droplet biogenesis at later stages of adipogenesis by providing DAG on the expanding droplets. Consistent with this hypothesis, lipin 1 expression levels influence droplet size and number in human macrophages (37) or hepatocytes during adaptation to hypoxia (38), and a recent report described that lipin inactivation in mouse adipocytes results in a multilocular phenotype (39). A requirement for lipins in droplet biogenesis may be also due to their roles in phospholipid metabolism. For example, altered membrane composition could influence ER structure and consequently the formation of droplets from the ER, or droplet dynamics by influencing their phospholipid monolayer (40). Remarkably, despite the efficient suppression of PAP activity, we did not find any significant alterations in the levels of the major phospholipid classes, suggesting that the changes in droplet biogenesis in the lipin 1/2-depleted cells are not depending on these pathways. Because PA has a potent inhibitory role in adipocyte differentiation (22, 39, 41), it is also possible that impaired adipogenesis prevents full maturation of the adipocytes, leading to fragmented lipid droplets. However, the facts that the PA-mediated inhibition takes place at the early stages of adipogenesis and that lipin 1 depletion has only a minor effect on total droplet volume per cell (Fig. 7*D*) imply a distinct later role of lipin 1 in lipid droplet biogenesis.

We showed here that depletion of lipin 2 either before or after initiation of 3T3-L1 differentiation has a positive effect on droplet biogenesis. A noteworthy difference between these two situations is the increase in adipogenic and lipogenic markers that takes place only when lipin 2 is depleted before initiation of differentiation. Overcompensation by one lipin in a background of another mutated lipin has been described previously in a variety of different models. For example, siRNA-mediated knockdown of lipin 2 in HeLa cells results in a compensatory increase of the mRNA and protein levels of lipin 1 and higher PAP activity levels (24). A recently described lipin 2-deficient mouse displays enhanced lipin 1 protein expression and PAP activity in the liver (35). Loss of lipin 2 in the shRNA-treated preadipocytes may therefore trigger a more potent differentiation through activation of lipin 1. Consistent with this hypothesis, overexpression of lipin 1β in 3T3-L1 cells results in increased expression of adipogenic markers and neutral lipid accumulation (8). On the other hand, PAP activity levels in the adipose tissue of the lipin 2-deficient mice are not different from those of the wild-type control (35). This may reflect differences between the cell and tissue types or the activation of PAP-independent mechanisms. Interestingly, although lipin 1 mRNA levels do not increase in the lipin 2 siRNA-depleted cells, lipin 1 protein levels did show an increase (Fig. 10*B*; 48% increase over control day 8 adipocytes in three separate experiments).

In summary, by characterizing an *in vitro* model with virtually no remaining lipin proteins after initiation of adipogenesis, we provide evidence for a requirement of lipins in lipid droplet formation. We also provide evidence for a negative role of lipin 2 in this process. An increasing number of lipin mutations have been described in mouse models and patients over the last few years. Exploring the pathways that underlie the co-regulation of lipin expression and activity is important to fully understand the phenotypes of these lipin deficiencies.

## Footnotes

3 The abbreviations used are:

PA: phosphatidate
PAP: phosphatidate phosphatase
DAG: diacylglycerol
TAG: triacylglycerol
PE: phosphatidylethanolamine
PC: phosphatidylcholine
PPAR: peroxisome proliferator-activated receptor
SVF: stromovascular fraction
LTDR: LipidTox Deep Red
qPCR: quantitative PCR.
